# Determinants of maternal role adaptation in mothers with preterm neonates

**DOI:** 10.5935/1518-0557.20200108

**Published:** 2021

**Authors:** Zahra Bostani Khalesi, Soheyla Mirzaii, Enayatollah Homaei Rad, Sepideh Panjalipour, Sodabeh Kazemi

**Affiliations:** 1 Social Determinants of Health Research Center, Guilan University of Medical Sciences, Rasht, Iran; 2 Student Research Committee, Guilan University of Medical Sciences, Rasht, Iran; 3 Reproductive Health Research Center, Department of Obstetrics & Gynecology, Al-Zahra Hospital, School of Medicine, Guilan University of Medical Sciences, Rasht, Iran

**Keywords:** Adaptation, Mothers, Role, Infant, Premature

## Abstract

**Objective:**

Becoming a mother is an innate process, without any culture-dependent instruction. While it is a pleasant experience, it can sometimes be associated with problems resulted from baby caring. Preterm birth can be a challenge for the maternal role adaptation (MRA). Therefore, the present study was conducted to determine the maternal role adaptation in mothers with preterm neonates.

**Methods:**

The present study is cross-sectional, with a sample including 114 mothers of preterm infants in the NICU. We collected the data using a two-section questionnaire. The first section was a demographic questionnaire and the second section was a standardized questionnaire? “Maternal role adaptation scale in mothers with preterm neonates admitted to neonatal intensive care units” (MRAS: NICU). We ran the statistical analysis using descriptive and inferential statistical methods with the SPSS 21 software.

**Results:**

The total MRA score was strong in half of the participants. The participants had a university education, were employed and satisfied with their economic status, and had a high score on adaptation to the maternal role. There are different domains to the MRA, the highest score was allocated to the participation in care (56.24±0.13), and the lowest score was allocated to growth and development (3.12±0.28).

**Conclusions:**

According to the results of this study, the most important factors associated with MRA are the mother’s age, education, and economic satisfaction. Determining the factors related to the mothers’ adoption of premature infants could increase the ability of mothers to cope with problems and negative emotions, and enhance the adoption of maternal roles.

## INTRODUCTION

One of the most important roles of a woman is the maternal role (Hadadi *et al*., 2011). Bearing a baby results in the acceptance of fundamental changes in cognitive, emotional, social, and behavioral functions (Bailey, 2010). This psychological change can be influenced by a woman’s specific circumstance, beliefs and attitudes, economic, social status, fitness, and knowledge, as well as social and psychological conditions, and as she is more mature, she will adapt better (Sayah & Hosaini Mojarad, 2011). Adaptation to the maternal role involves conceptualizing and establishing a responsible maternal role that is recognized by the creation of a new identity and the formation of maternal behaviors (Kearvell & Grant, 2010). Generally, the transition to the maternal stage as a natural crisis presents significant adoption problems for most mothers, and is a significant issue for health care providers (Bailey, 2010). When the preterm birth occurs, additional stress adds to this crisis, which is exacerbated by the admission of the infant to the NICU (Holditch-Davis, 2007).

Preterm and low birth weight infants have needs that require intensive neonatal care. These infants not only require intensive care immediately after birth, but they also have a higher morbidity rate (Morey & Gregory, 2012). The mothers of these infants are at risk for maladjustment (Heidari *et al*., 2013). The results of studies show that mothers of preterm infants have more difficulty than mothers of term infants in adjusting to their maternal roles (Lee *et al*., 2009; Forcada-Guex *et al*.,2011; Gogate, 2020). In the case of preterm birth, the process of adjusting to the developmental crisis of having a new infant and the situational crisis of having a preterm infant provides a variety of stressors for the new mother (Pyhälä *et al*., 2009). Hospitalization of the infant is a stressful issue that causes disorder in the emotional and interdependent association between mother and infant (Dashevsky, 2012). A mother’s ability to adjust to the stressors is related to the nature of the stimuli and to the mother’s existing mode of adaptation. In other words, the more preterm the infant, the more intense the stressors that affect the mother’s existing mode of adaptation (Gogate, 2020). A mother whose infant is admitted to the NICU has limited opportunities to care and interact with the infant, and this delays the acquisition of the maternal role (Rajabi *et al*., 2018). Of course, previous studies have shown that maternal role adaptation is influenced by a number of factors, such as social support, prejudice (Khandan *et al*., 2018), and judgmental prevention, self-efficacy (Niknajad *et al*., 2012), and culture (negative reactions from the community) (Heydarpour *et al*., 2017).

Berkowitz (2005) also indicated that the maternal role in mothers of premature infants admitted to NICU, not only is influenced by the maternal social communication, but also affects it, and it seems there are still many unknown aspects about it that need more investigation.

Since successful adaptation to the maternal role results in self-esteem and the satisfaction of her abilities in caring and nurturing the child; and awareness of its related factors in mothers of preterm infants admitted to the NICU can improve the quality of postpartum care (Shin, 2004). The researchers decided to conduct a study aimed at determining the extent of maternal role adaptation in mothers with premature infants and its related factors.

## MATERIAL AND METHODS

The present study is a descriptive analysis. The participants were 114 mothers of premature infants admitted to the NICU. The inclusion criteria included consent to participate in the study, reading and writing skills, aged 18 and over, without any mental disorders, living with spouse, first marriage, first maternal experience, single infant, premature (Preterm child born in 28-36 weeks gestational age), without any apparent abnormalities, and the reluctance of the mothers to continue participating in this study was the exclusion criteria.

The necessary information regarding the objectives of the study was explained to each participant. All participants signed a written consent, and their confidentiality and privacy were maintained. In addition, we considered ethical principles in data gathering and analysis.

Data were collected using a three Self-reported questionnaire, including demographics, the standardized tool: “Maternal role adaptation scale in mothers with preterm neonates admitted to neonatal intensive care units” (MRAS: NICU) and related factors. The demographic questions consisted of items regarding age, education, ethnicity, occupation, average monthly income, alcohol, drug use and smoking.

MRAS: NICU was developed by Heidarpour et al. The MRAS: NICU is a 32-item-scale, designed to measure maternal role adaptation. This scale consists of 6 domains, including participation in care (14 questions), self-efficacy (6 questions), distant mothering (3 questions), uncertainty (4 questions), Interaction (3 questions), growth and development (2 questions) that correspond to a 5-point Likert scale, ranging from “1” Strongly Disagree to “5” Strongly Agree. The score of each domain was calculated as the sum of the scores of the items in that domain, and the total score of the scale was calculated as the sum of the domain scores, then the scores were converted to percentages and classified in three levels: Poor (0-33%), moderate (34-66%) and strong (67-100%). The mean for the full scale was used where the scores ranged from 1 to 5. The higher scores indicated higher maternal role adaptations. The instrument reliability with Cronbach’s alpha coefficient of 0.90 and its stability after re-test with correlation coefficient of 0.81, were confirmed by Heidarpour *et al*. (2016).

Maternal role adaptations related factors included maternal factors (satisfaction of living with spouse, satisfaction with economic-welfare status, duration of marriage, participation in childcare, family and friends support for child care, family structure, awareness of the pregnancy process, childbirth, and parenting roles); paternal (Age, Ethnicity, Education, Occupation, Average Monthly Income); and infant factors (Age of Birth, Sex, Satisfaction of Child Sex, Birth Weight), Fertility history (planning for birth, infertility history, abortion history, and fetal death).

After data collection, data was inserted in SPPS version 21, for determining the score of the” Maternal Adaptation” questionnaire as a whole, and we divided the domains’ statistical indices including mean, mode, minimum, maximum and standard deviation.

## RESULTS

The mean age of mothers and their husbands was 29.34 (SD=6.06) and 31.11 (SD=6.91) years old, respectively. Most of the mothers were housewives (60.5%) and 40.4% were college graduates (40.4). Most of the fathers were self-employed (50.4%) and had a Diploma (45.6%).

As far as ethnicity of mothers is concerned, 94.73% were pf Guilanian ethnicity and 60. 5% were housewives. 52.4% of the mothers were satisfied with their economic status. In case of neonatal sex, 55.26% had boys and 69.29% planned their pregnancy.

Table 1 shows the demographic characteristics of the participants.

**Table 1:** Demographic Characteristics of the Study Population

Variable		Result
Mother’s age (µ±SD)		29.34±6.06
Father’s age (µ±SD)		31.11±6.91
Ethnicity (%,n)	Guilanian Non Guilanian	94.7(108/114) 5.3(6/114)
Mother’s job (%,n)	Housewives Laborer Employee Farmer Self- employed	60.5 (69/114) 3.5 (4/114) 14.0 (16/114) 6.2 (7/114) 15.8 (18/114)
Father’s job (%,n)	Unemployed Laborer Employee Farmer Self- employed	2.6 (3/114) 14.0 (16/114) 22.8 (26/114) 10.6 (12/114) 50.0 (57/114)
Mother's education (%,n)	Illiterate Primary school Secondary school Diploma University	6.1 (7/114) 7.9 (9/114) 12.3 (14/114) 33.3 (38/114) 40.4 (46/114)
Father’s education (%,n)	Illiterate Primary school Secondary school Diploma University	6.1 (7/114) 4.4 (5/114) 9.6 (11/114) 45.6 (52/114) 34.3 (39/114)
Smoking (%,n)	Yes No	5.3 (6/114) 94.7 (108/114)
Economic satisfaction (%,n)	Yes No	52.4 (63/114) 47.6 (51/114)

The mean MRAS score: NICU was 118.77±3.31. MRA in 45% of mothers was strong, 23% moderate and 32% poor.

In the six MRAS sub-scales: NICU, the mean scores in Participation in care (M=56.24, SD=0.13); Self- efficacy (M=23.2, SD=1.02); Maternal distance (M=11.34, SD=0.22); Uncertainty (M=14.61, SD=1.41); Interaction (M=10.26, SD=0.25); Growth and development (M=3.12, SD=0.28). *Overall*, the highest score of six sub-scales was Participation in care (80.34%); and the lowest score was Growth and development (52%) (Table2).

**Table 2:** Distribution of MRAS: NICU total and sub-scales

MRAS: NICU Sub-scale	Range of Scores	Mean	Standard deviation
Participation in care	14-70	56.24	0.13
Self- efficacy	6-30	23.2	1.02
Distance maternal	3-15	11.34	0.22
Uncertainty	4-20	14.61	1.41
Interaction	3-15	10.26	0.25
Growth and development	2-6	3.12	0.28
Total score	32-160	118.77	3/31

Table 3 shows the findings of the regression model in relation to investigating the association between different variables and the MRAS: NICU score.

**Table 3:** Coefficients of Linear Regression Model of MRA according to selected variables

Socio- individual variables	Coefficients		*p* -value	Coefficient of Confidence Interval 90%	
	Coefficient	Standard deviation		High	Low
Fixed value	27.58	0.11	0.00	38.54	16.61
Mother's age	-0.016	0.04	0.02	0.06	-0.03
Mother's ethnicity	-1.06	1.4	0.44	1.71	-3.85
Mother's education	-0.12	0.03	0.01	0.16	-0.02
Mother's job	0.24	0.2	0.23	0.65	-0.16
Smoking	1.27	1.43	0.37	4.11	-1.56
Economic satisfaction	-0.21	0.16	0.02	0.5	-1.05
Participation in care	1.03	1.04	0.52	8.73	-4.51
Father's age	-0.03	0.08	0.64	0.13	-0.2
Father's education	-0.25	0.4	0.52	0.55	-1.06
Father's job	0.33	0.32	0.29	0.97	-0.29

According to the T-test, the gender of the infant (female or male), the Smoking (yes or no), mother’s and ethnicity (Guilanian or Non-Guilanian) and according to the Analysis of Variance (ANOVA) test, father’s job (unemployed, laborer, employee, farmer, self- employed) and mother’s job (housewives, laborer, employee, farmer, self-employed) did not have a significant effect on the mean MRAS score: NICU. In addition, there was a significant correlation between the MRAS score: NICU and the mother’s age, education, and economic satisfaction were significantly related to the MRA (Table 3). 

## DISCUSSION

The results of this study showed that mothers with higher education had higher maternal role adaptation scores. According to the results from the Heydarpour study, lack of adequate information on what happens after delivery following preterm birth is confusing to mothers (Heydarpour *et al*., 2017). Lee et al. concluded that the lack of knowledge of premature infant care is the most important challenge for mothers (Lee *et al*., 2009). *Another study by* Horst et al., reported that *having knowledge* and skills to care for mothers *with premature babies* was the first priority for these mothers (Hurst, 2001). Since *mothers’ educational* levels were significantly associated with the increased *knowledge* on premature childcare, the findings of this study are consistent with other similar studies.

The results are indicative of the fact that employed mothers had a higher maternal adaptation score. Rezaei (2010) stated that employed women had higher self-esteem, which made coping with difficult conditions easier for them. In another study, Dashevesky considers a lack of self-esteem as a contributing factor to reduce maternal competence feelings (Dashevsky, 2012). Increasing self-esteem leads to greater satisfaction with the maternal role (Heidari *et al*., 2012). The obtained results are consistent with those from other studies.

The results of this study showed that mothers who were satisfied with their economic status had higher maternal role adaptation scores. According to the results from the study by Holden et al., a better economic status will increase the self-efficacy of preterm infants’ mothers (Holden *et al*., 2018). The results of the Heydarpour study also showed that self-efficacy is among the main elements resulting in adaptation to the maternal role (Heydarpour *et al*., 2017). Mothers, who have higher self-efficacy, have an easier transition to the maternity phase, thereby causing their general health to be less exposed to threats (Russell, 2006). The results of this study are in line with those from the stated studies.

According to the results on the score of adaptation to the maternal role by domains, the highest score relates to maternal participation in childcare, and continued confidence accounts for the lowest score. Most of the mothers whose infants are admitted to intensive care, have to share their maternal role with nurses and other caregivers, and their opportunities for infant care are limited (Zareinejad *et al*., 2018). Since one of the important aspects of the infant-mother communication process is the active participation of the mother in the care of the infant, we must encourage, support mothers to begin taking care of their infants, and eventually take full responsibility for them (Bialoskurski *et al*., 2002). Results from the study by Ben-Ari et al. showed that mothers who did not play an active role in caring for their infants had a reduced maternal capacity to get adapted to maternal roles (Taubman-Ben-Ari *et al*., 2009). In the intensive care unit, mothers are often away from their babies, which, in turn, increases the mother’s stress, whereas maternal support reduces stress (Neri *et al*., 2015). So, not only should nursing care focus on the treatment of the infant but also on the emotional support for the mother (Dezvaree *et al*., 2016). Meanwhile, the support of nurses and families increases the mother’s self-confidence and suppresses her negative emotions (Khandan *et al*., 2018). Thus, nurses who are working in the neonatal intensive care unit should support and assure mothers to move into the maternity phase sooner.

## CONCLUSION

Adaptation to the maternal role is a phenomenon that starts during pregnancy and continues after delivery. Mothers with preterm labor, face disorders in this process. Among the various factors, the mother’s age, education, and economic satisfaction, increase adaptation to the maternal role. In so doing, providing appropriate information to mothers, social and emotional support for them, and through proper interaction between nurses and physicians with these mothers, an appropriate step can be taken to design effective interventions to increase adaptation to the maternal role in this group.

Due to the complexity of adaptation in parenthood, a mixed-method, including a quantitative and qualitative study is recommended, to determine the factors affecting MRA.
